# The Effect of Weld Reinforcement and Post-Welding Cooling Cycles on Fatigue Strength of Butt-Welded Joints under Cyclic Tensile Loading

**DOI:** 10.3390/ma11040594

**Published:** 2018-04-12

**Authors:** Oscar Araque, Nelson Arzola, Edgar Hernández

**Affiliations:** 1 Department of Mechanical Engineering, Universidad de Ibagué, Ibagué 730001, Colombia; 2 Research Group in Multidisciplinary Optimal Design, Department of Mechanical and Mechatronics Engineering, Universidad Nacional de Colombia, Bogota 111321, Colombia; narzola@unal.edu.co (N.A.); edhernandezl@unal.edu.co (E.H.)

**Keywords:** butt weld joint, fatigue, crack growth rate, weld reinforcement, cooling rate

## Abstract

This research deals with the fatigue behavior of butt-welded joints, by considering the geometry and post-welding cooling cycles, as a result of cooling in quiet air and immersed in water. ASTM A-36 HR structural steel was used as the base metal for the shielded metal arc welding (SMAW) process with welding electrode E6013. The welding reinforcement was 1 mm and 3 mm, respectively; axial fatigue tests were carried out to determine the life and behavior in cracks propagation of the tested welded joints, mechanical characterization tests of properties in welded joints such as microhardness, Charpy impact test and metallographic analysis were carried out. The latter were used as input for the analysis by finite elements which influence the initiation and propagation of cracks and the evaluation of stress intensity factors (SIF). The latter led to obtaining the crack propagation rate and the geometric factor. The tested specimens were analyzed, by taking photographs of the cracks at its beginning in order to make a count of the marks at the origin of the crack. From the results obtained and the marks count, the fatigue crack growth rate and the influence of the cooling media on the life of the welded joint are validated, according to the experimental results. It can be concluded that the welded joints with a higher weld reinforcement have a shorter fatigue life. This is due to the stress concentration that occurs in the vicinity of the weld toe.

## 1. Introduction

In comparison with other methods for the joint of materials, welding is one of the most used ones. However, it holds diverse flaws related to the manufacturing process. The most common imperfections are discontinuities, porosities, undercuts, cracks, inclusions, and lack of fusion, among others. These flaws are produced by the increase of speed, change in polarity of the current, and the extension and diameter of the electrode. Additionally, these variables modify the grain size and the areas surrounding the weld bead, such as: the fusion zone (FZ), the heat affected zone (HAZ), and the base metal (BM) [1].

An evaluation of fatigue strength for welded joints consist of the study of crack initiation and propagation phenomena. Due to this, it is necessary to know the mechanisms to determine the various typical factors of fracture mechanics, with the aim to explain the behavior of cracked material when subjected to stresses and the mechanisms that lead to premature failure in load conditions below the yield and breakage limits [2]. The fracture mechanics shows that the parameters tenacity, crack size, and level of strength can be related to predict the possibility of a fracture, such as stress intensity factors (SIF) in butt welds [3,4,5]. This parameter depends on the geometry and the load and it is a measure of the degree to which a load is amplified at the tip of a crack. Additionally, the fracture mechanics raises other expressions for the evaluation of the crack propagation rate (da/dN), as function of the SIF range (∆K), where C and m are material constant values. This relationship is known as the Paris law [6].

(1) $$\frac{\mathrm{da}}{\mathrm{dN}}=C{\left(\Delta K\right)}^{m}$$

Several researches have been developed aimed at determining the areas that make up the welded joint [7,8,9]. They have generally identified the fusion zone (FZ), which presents a grain growth from the fusion line into the interior of the fusion zone, the heat affected zone (HAZ) characterized by the hardening during the heating. In the cooling, the microstructure can present evidences of liquefaction in edges of grain and the base metal (BM), corresponding to the formation limit of the hardener precipitate. In the research of [10], there are observed microstructural variations in a series of low-alloy steel weld which deposit different carbon concentrations (product from experimental electrodes), getting allotriomorphic ferrite growth is assumed to occur by an equilibrium transformation mechanism; its formation is found to determine the development of both Widmanstätten and acicular ferrite.

The observation of the fault surfaces in welding are useful to estimate the mechanisms of crack propagation, using the mark counting. In the research of [11], it has been observed that the marks are normally formed when the load is intermittent during the service and the marks in a much finer scale. They can show the tip position on the crack after each cycle. The observation of the marks always suggests a fatigue failure, but the absence of this pattern does not rule it out. The crack origin and, in some cases, the marks can be observed through a scanning electron microscope (SEM). In the work of [12], the modeling and implementation of criteria for multiple crack propagation, including interaction and coalescence was made. In this research, the correlation between the number of initiation sites and the applied stress level, showing that crack propagation is related with fatigue strength of weld. 

The determination of the mechanical properties of the weld joints such as hardness, fracture toughness, yield strength, and tensile strength are not constant, Bullón et al. [13] indicates that they are affected by the thermodynamics or thermal field (temperature field) due to the high gradient temperature changes that are generated causing microstructural transformations, which affect the mechanical field (field of stresses and strain) and metallographic field (field of microstructural state) causing morphological changes. In addition, there is a mutual influence between this fields represented by the continuous and discontinuous lines.

Other researchers have studied the effect of microstructural changes and mechanical properties on the welded joint. Various researchers [14,15] show that geometric discontinuities affect the fatigue strength of welded joints. It is in the HAZ where the discontinuities can nucleate, which in general are the initiators of fatigue cracks in the welded joints. The fatigue fissure begins in a local flaw of the structure, either internal or external. In fact, the bead geometry already constitutes a geometric discontinuity, which turns out to be a stress concentrator.

In regards to the analysis by fracture mechanics on welded joints, several authors have researched the topic over the years through the selection of a combination of factors, such as material, the model geometry, and load conditions. However, most researchers have opted for the use of computational tools and the finite element method (FEM). A study using this technique by the Berrios’ researcher [16], found a remarkable correspondence between the experimental trajectories of crack propagation on high resistance-steel and the propagation trajectories obtained using the software ANSYS APDL. The research carried out by Araque and Arzola [17], developed an experimental-theoretical analysis about the influence of the cooling medium and the geometry of the welding bead profile in fatigue life and the associated parameters with structural integrity of welded joints. The results were a set of analytical expressions for the weld magnification factor Mk, the obtained models of behavior of the weld magnification factor are compared with the results from other researchers with some small differences.

On the other hand, Toribio [18] has identified life fatigue for a pressure vessel under cyclic loads of thermal origin, as well as proposal on a general procedure to calculate the life fatigue on any structure subjected to cyclic load. He has been determined that shorter fracture trajectories during the life service generate leaks prior to break.

On the same field of research, Lewandowski [19] has analyzed the behavior of crack growth in ferritic-perlitic structures subjected to cyclic flexion and has determined that, in all cases, life fatigue on welded specimens is less than on completely solid specimens, due to different mechanical properties to the characteristics of the welded joints (base material, weld bead, HAZ thermal affectated area).

About the use of software to analyze fractures, Franc3D has gained popularity among researchers in the last decade. Yang [20], has proposed an algorithm based on the linear elastic fracture mechanics for the simulation of fatigue crack growth under non-proportional loads. He has found that cycles of growth calculated on the basis of stress intensity factors (SIF) correspond to the experimental data for specimens subjected to slightly lower loads. Similarly, Xiao [21] has made a numerical analysis for surface cracks in on a hemispherical hole and has concluded that the geometry of the whole surface is a determining factor in the SIF. By using this software, researcher Chin [22] has estimated how a crack spreads on a support made from an alloy of titanium and aluminum (very common in the aerospace industry) and has found that the Type I (SIF’s) is below the fracture toughness to the fracture material. Then, he has concluded this support could tolerate a certain type of cracks.

This article shows an experimental and theoretical study of the fatigue failures on butt welded joints for structural carbon steel ASTM A36 HR that used shielded metal arc welding (SMAW) with electrode E6013. The influence of the weld reinforcement was evaluated, as well as the post weld cooling environment by the uniaxial fatigue tests and the failure mechanism analysis present in the fracture surface. In other tests, the mechanical and fractomechanical properties from the welded joints were analyzed in relation with the crack origin and propagation caused by fatigue.

## 2. Materials and Methods

This section can be divided by subheadings. It should provide a concise and acute description of the experimental results, their interpretation as well as the experimental conclusions that can be drawn.

### 2.1. Experimental Design

For the uniaxial fatigue tests, 8 replicates were selected for each treatment accounting for a total of 32 test specimens. Two experimental factors were established: weld bead geometry and the post-welding cooling media. Two levels for each factor the welding reinforcement (1 and 3 mm), and the cooling rate, slow (quiet air) and fast (immersion in water) are evaluated, respectively. For the Charpy impact energy test six replicates for each treatment were carried out, 3 in the (FZ) and 3 in the (HAZ), respectively. Moreover, 3 more replicates were analyzed for the base metal (BM), thus 27 test specimens were obtained for the late type of test. Finally, for the uniaxial tension test three samples were evaluated, a total sampling amount for each treatment, accounting a total of 12 test specimens. To make the data management more feasible a codification system was assigned for each test type and cooling medium, correspondingly. This can be found in Table 1 for the uniaxial fatigue test specimens, Table 2 for Charpy impact energy test specimens and, finally in Table 3 for the tension test specimens, respectively. For example, 1F-A code is the test specimen with 1 mm of welding reinforcement, cooled in water and is the replica A.

### 2.2. Materials

Rolled carbon structural steel ASTM A36 with a plate thickness of 6 mm was used on the welded samples for the uniaxial fatigue and uniaxial tension. For the Charpy impact energy test a plate thickness of 12 mm was used. The mechanical properties for ASTM A36 are shown in Table 4. The chemical composition of the material was checked by spark spectrometry test UV-VIS (Shimadzu Scientific Instruments, Kyoto, Japan) and the results are presented in Table 5.

### 2.3. Welding Procedure

The E6013 electrode is used as a filler for the welded joints. The shielded metal arc welding process SMAW was used for welding procedure, the plane to top position was utilized with a butt welded joint design following, voltage ranging between 15–42 V and current between 85–140 A DC. The welding was performed perpendicularly to laminar direction of BM. Linear speed between 0.12 and 0.18 m/min. The applied weld passes depended on the over-thickness width, as follows: single pass for 1 mm over-thickness and two passes for 2 and 3 mm over-thickness, respectively. The welding process was carried out following a standard SMAW procedure and executed by a qualified welder [23]. The flaws found in the welded joints were within what was expected in this type of joints [24]. Subsequently the plates were cut using abrasive waterjet (AWJ) method.

### 2.4. Uniaxial Fatigue Test

**Figure 1:**
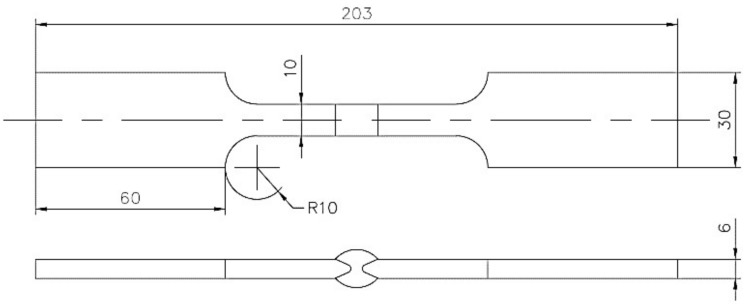
Diagram of tensile and fatigue test specimens (mm).

The same geometry was used for the fatigue and uniaxial tension test specimen. In Figure 1 a diagram for the experimental sample is shown. The AWS B4.0:2007 and ASTM E606 standards were considered for the specimen configuration and design. 

**Figure 2:**
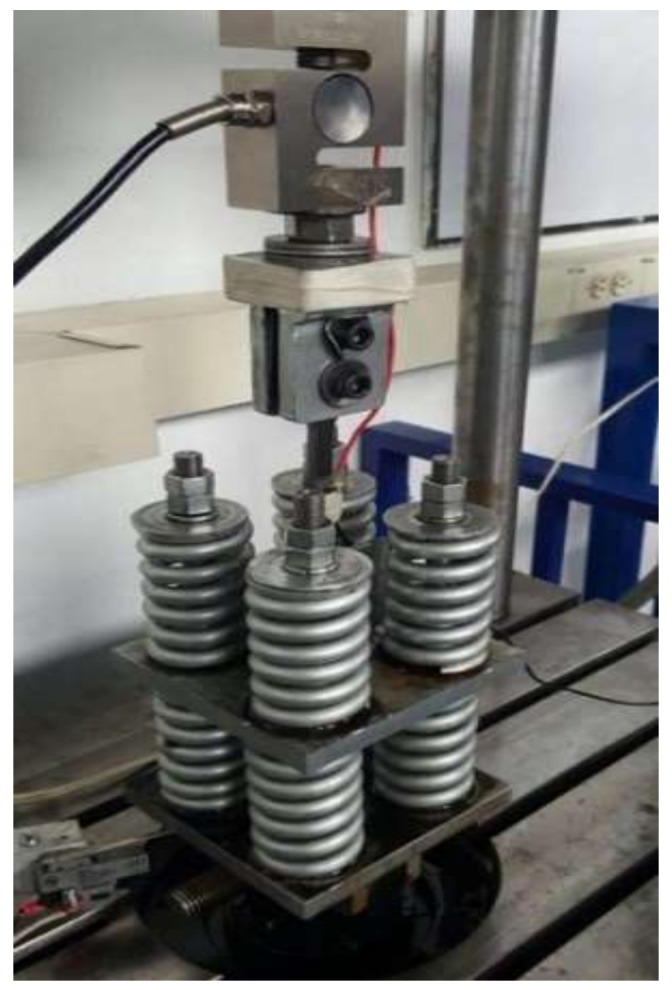
Specimen mounted on the uniaxial fatigue machine.

Subsequently to post-welding procedure, the final geometry of specimens was achieved with a waterjet cutting machine to avoid modifications on post-welding treatment. For uniaxial fatigue experimental test, a non-standard test machine was used as shown in Figure 2. The parameters for the fatigue test used were: frequency of cycle load 4 Hz, peak load 12 kN and a load cycle ratio 0.05, respectively. A load cycle ratio R > 0 is fixed, to guarantee non-compressive contact between the crack surfaces and then avoid loss of information regarding the propagation rate.

### 2.5. Uniaxial Tension, Microindentation Hardness, and Charpy Impact Energy Tests

**Figure 3:**
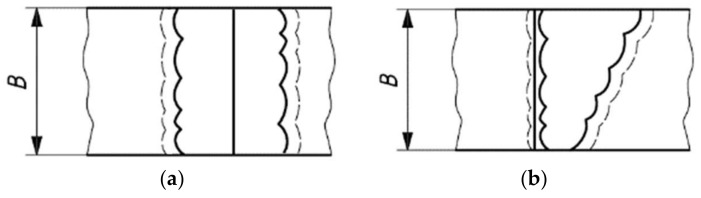
(**a**) Notch location in the fusion zone (FZ) and (**b**) the heat affected zone (HAZ), according to the Standard ISO 15653.

The uniaxial tension tests were performed in a universal test machine Shimadzu UH-500 kNI (Shimadzu Scientific Instruments) following the standard ASTM E8. These tensile tests were carried out with the purpose of obtaining the tensile mechanical properties of the samples studied and finding possible differences between the studied treatments. The micro-indentation hardness test was performed in relation with sample thickness (6 mm) on three equidistant lines and a third of the sample thickness, employing a durometer LECO M-400-G2 Hardness Tester (LECO Corporation, St. Joseph, MI, USA), with a load of 500 g during a time of 10 s (HV 0.5). Specimen fabrication for the impact energy characterization test, its geometry and percentage of fracture due to shearing were carried out by following the standard ASTM A370. The tests were performed at environmental temperature 19 ± 0.1 °C. A macro attack was performed with Nital at 3% over the welding profile following standard ISO 15653. Care was taken to locate the notch in the center of zones HAZ and FZ as shown in Figure 3.

### 2.6. Microstructure and Fractography

The microstructural characterization of the welding zones was performed by SEM (Quanta 200-r, FEI, Hillsboro, OR, USA) and optical microscope (E600, Nikon, Tokyo, Japan). Likewise, the analysis of failures over crack surface was performed on the failed specimens, to identify the cracks origins, planes of crack stable propagation and final fracture. Through observation with stereoscope, the stable propagation crack zone to readily use the SEM. This last technique was used to counting striations procedure, close to initiation and near the end of stable propagation crack zone, respectively. Details on how to perform this procedure can be found on the study carried out by DeVries et al. [25].

### 2.7. Crack Growth Modelling

The procedure used in this work, looking forward to obtaining an appropriate numerical model, consisted on the following phases: creation of the model in CAD software (Autocad 2018, Autodesk, CA, USA); residual stresses analysis; field analysis of the stress-strain before the crack growth, incorporation and crack growth and finally, the construction of Paris’ curve from the stress intensity factors calculated by the software (FRANC3D) [26].

**Figure 4:**
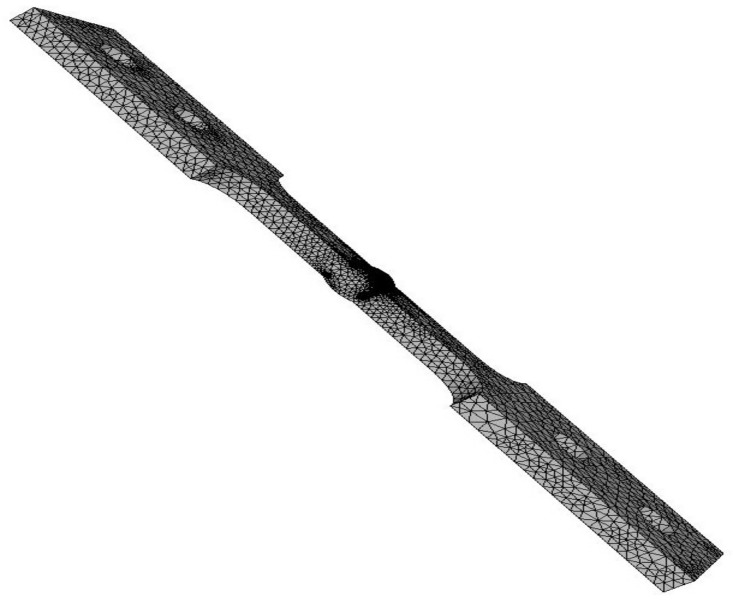
Detail of the mesh generation for the weld joint model.

The model is generated in the 2016 Solidworks CAD software and then imported into the software ANSYS APDL (V17, Ansys Inc, Canonsburg, PA, USA). A preliminary modeling of finite elements of the welding joint model was carried out, taking as a goal a fatigue finite life, thus finding a peak stress of 192 MPa. The final model is loaded with a peak stress tension of 192 MPa on the top, and at the opposite end a built-in restriction type is placed. Furthermore, a linear elastic and isotropic material with a 200 GPa elastic modulus and Poisson coefficient of 0.26. In Figure 4, there is a detail of the meshed weld bead.

Simulations to get the residual stress profile, due to the thermal cycle welding process, were carried out on finite element software Ansys 17.2 (Ansys Inc.). These add-ons were Steady-State Thermal, Transient Thermal and Transient Structural. The utilized add-on in this case in non elasto-plastic. It required a set of mechanical and thermal properties for the base material and the welded join, the convective cooling behavior, percentages on each phase transformed, as a function of temperature and thermos-dynamic coupling. The aim of this study is to get the residual stress field in a transversal direction to the weld beat. This stress field is over placed to fluctuating fatigue stresses, so that there can be a modeling of the stress intensity factor (SIF). 

**Figure 5:**
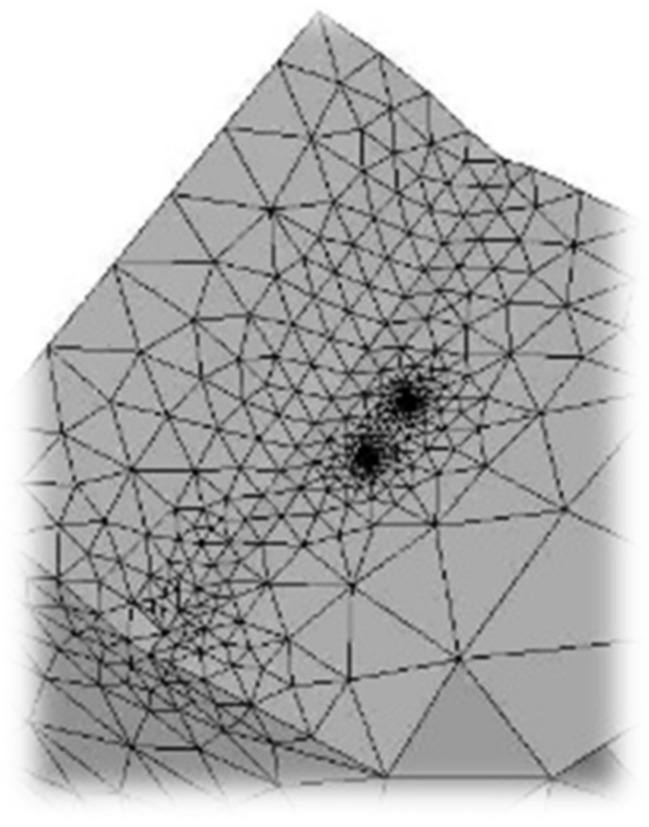
Inserted semielliptical surface crack at the weld toe (FRANC3D).

In the model, the location of the initial micro-crack corresponds to one of the selected specimens tested experimentally. The location corresponds to a 20% eccentricity with regards to the average plane on the test piece, at the weld toe. A semielliptical surface crack was used. Once the location and crack geometry are defined, a type-CDB ANSYS file is generated. The latter should be compatible with FRANC3D software. After the import of the model into this software, the crack is inserted (see details of the crack in Figure 5), and the propagation simulation starts, according to well-defined grow aspects of stable growth (growth model, size, number of iterations, etc.).

In Table 6, different semielliptical surface crack sizes used on every interaction are shown. The aspect ratio of the crack remains constant between one iteration and the next. 

Finally, once all the iterations are run, the next step is extract the information from the SIF’s (stress intensity factors), and then build the respective Paris’s curves. The procedure described above was performed for test specimens whose thickness on the weld bead corresponded to 1 mm and 3 mm, respectively.

## 3. Results and Discussion

### 3.1. Overall Experimental Fatigue Behavior

**Figure 6:**
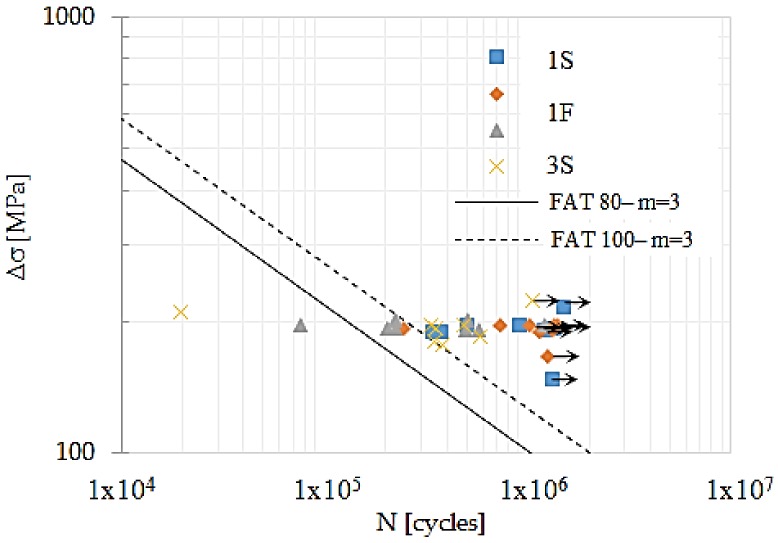
Experimental data and S-N curves according with classes FAT 80 and 100.

Using classes FAT 80 and 100 as a reference, the comparison between results for fatigue of butt welded joints for the analyzed treatments are showed in Figure 6. It is appreciated that, treatment 3S shows 50% of its data under limit of class FAT 100. Further, it is seen that only one sample of treatments 3S and 3F are under limit of class FAT 80, while mostly there are data for samples 1F and 1S. The latter represent a major deviation above limit of class FAT 100. Considering these partial results, it is necessary to perform a statistic evaluation for a better interpretation of the results. Based on [3] a statistical procedure to evaluate surviving probability as function of fatigue capacity (log C), which is obtained through (2):

(2) logN=logC−m·logΔσ

where for steels m = 3.

**Figure 7:**
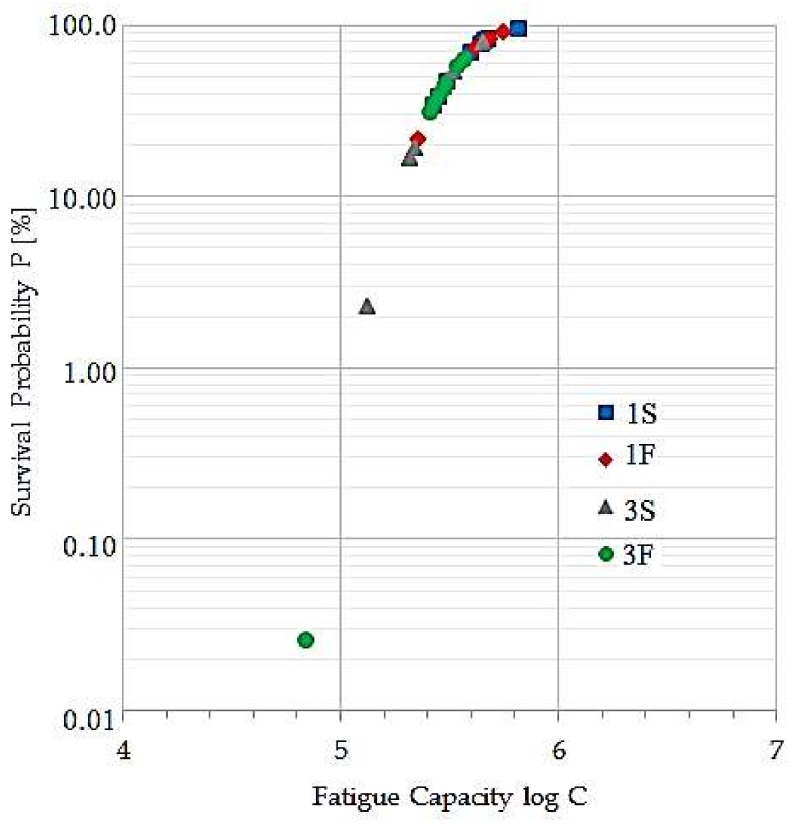
Survival probability for the butt welded joint specimens.

Subsequently, the surviving probability plot for the 32 samples is obtained. The latter is presented in the Figure 7, where it is possible to identify the behavior of welded samples in comparison with Figure 6. In Figure 7, it is observable that, in treatment 1S approximately 63% of the samples are above the 70% of surviving probability. The remaining are between 30% and 50% of surviving. In the treatment 1F, the 87.5% of the samples are between the 60% and 90% of surviving and remaining in 19%. In treatment 3S, the 50% of the samples are between 40% and 80% of surviving and the other 50% of the samples are between 2% and 20% of surviving. Finally, in the samples of treatment 3F, the 87.5% of the samples are between 30% and 70% of surviving and remaining in 0.025%. Thus, it is possible to state that treatment 1F is the one which has the highest surviving probability in comparison with the other treatments. On the contrary, the treatment 3S shows the lowest probability of surviving.

### 3.2. Crack Growth Model

#### 3.2.1. Convergence Analysis

A computational cost and result variations assessment is carried out for the finite element models, as it should do with the processing time and the relative error. Taking the stress intensity factor (K_I_) as a reference, as calculated according to Anderson [27], the input parameters for the computational analysis are larger radii than 1.25 mm, the smaller radii than 0.50 mm, with thickness of 1 mm. The results obtained for each numerical model are shown in Table 7.

**Figure 8:**
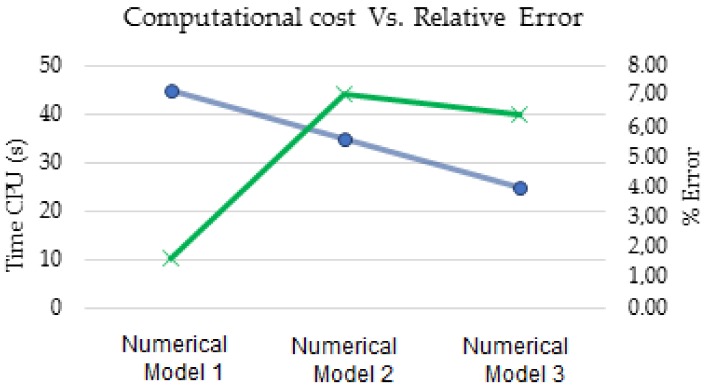
Computational cost vs. relative error.

Bearing in mind Figure 8, it can be seen the break-even point between time and central processing unit (CPU) and the error percentage was about the same as the Numerical model 2, which made the selected mesh-type for simulations.

#### 3.2.2. Results to the Propagation Crack Model 

**Figure 9:**
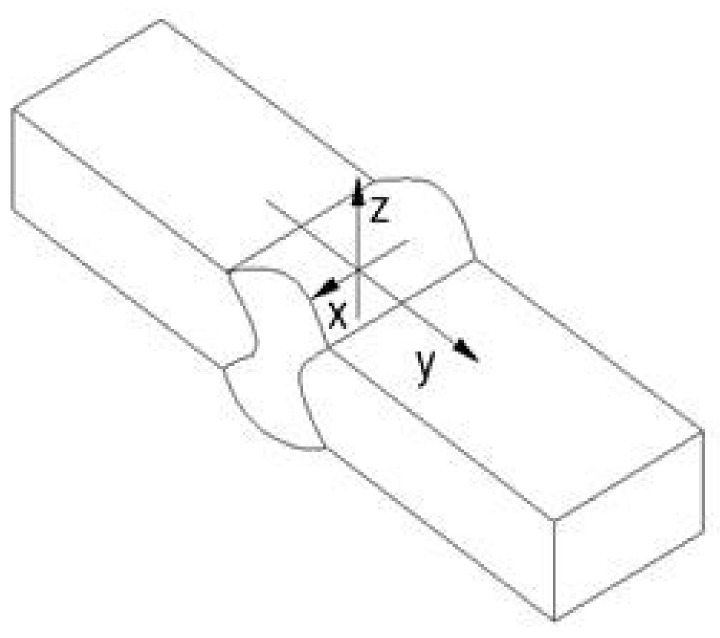
Nomenclature for directions relative to weld: longitudinal (x), transverse (y), and through-thickness (z) respectively.

**Figure 10:**
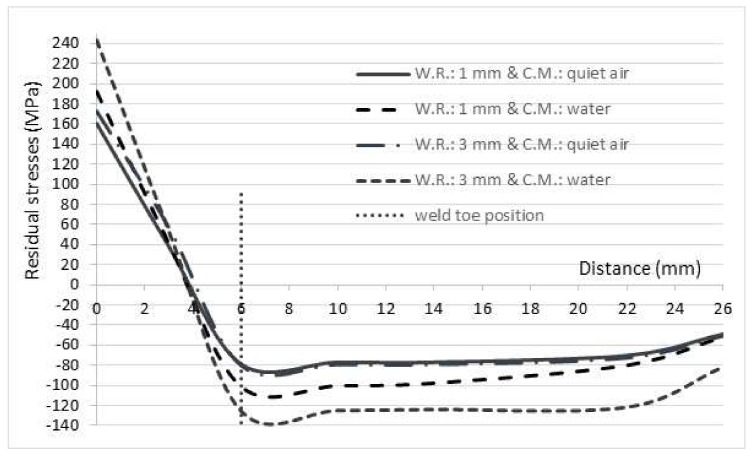
Residual stress profile on transverse direction to weld beat.

Figure 9 shows the convention used in this paper for describing the residual stress directions longitudinal (x), transverse (y), and through-thickness (z), respectively, relative to a real profile of a butt-welded joint. The residual stresses found in the three directions are different. Residual stresses in the longitudinal and through-thickness directions were smaller than transverse residual stresses. This is caused by the different restraints acting in the three directions. Moreover, transverse residual stresses are the important ones, due to the longitudinal orientation of the superficial semielliptical crack implemented in this study. In Figure 10, the behavior of transverse residual stresses can be seen for the 1 mm and 3 mm weld reinforcements (W.R.), and quiet air and water as cooling media (C.M.). For the four treatments, the resulting residual stresses are compressive for the weld toe position and diminish rapidly after one position about 22 mm far from the weld centerline. It can be concluded that the effect of the welding reinforcement on the residual stresses is weak when quiet air is used as cooling medium. The difference in the residual stresses is small between the quiet air cooling medium treatments; an average of 2 MPa greater for 3 mm of weld reinforcement is observed when compared with 1 mm of weld reinforcement. However, a much more marked influence of the cooling medium on the residual stresses is observed. The treatment with levels of 3 mm for the weld over thickness and water as a cooling medium reaches compressive residual stresses of −125.5 MPa for the position of the weld toe.

In this work, an experimental verification of residual stresses is not performed; although it is possible to make certain comparisons with the results obtained by other authors. For example, researcher Knoedel and colleagues [28] develop a phenomenological numerical model, useful in principle for any type of welded joint of steels after adjusting the process parameters and the properties of the materials dependent on temperature. Their results are also validated with experimental data from other previous works, finding a reasonable similarity. Using this phenomenological model, they find a profile of residual stress similar to that found in the current work for a welded joint of HT-36 steel plates. As a main difference, it appears that in the current work the residual stresses are compressive for the position of the weld toe, while in [28] the residual stresses for this location are tension, decreasing rapidly and moving to compresses in a position a little more away. The differences found are not surprising, given that it is widely known that both experimental measurements and the results obtained from numerical models are often subject to great variability and dispersion [28,29]. The above product, the large number of factors of the welding process, and properties of the materials involved cannot be completely controlled. Additionally, it is difficult to find experimental arrangements in framework that coincide with respect to the materials and procedures used.

Other authors [30,31] have reported that residual stresses are progressively relaxed due to the cyclic plastic deformation characteristic of the fatigue phenomenon. However, in this work, the field of residual stresses was imported into the model for the determination of SIF and remained constant throughout the process of crack growth. This consideration should be re-evaluated for future work.

**Figure 11:**
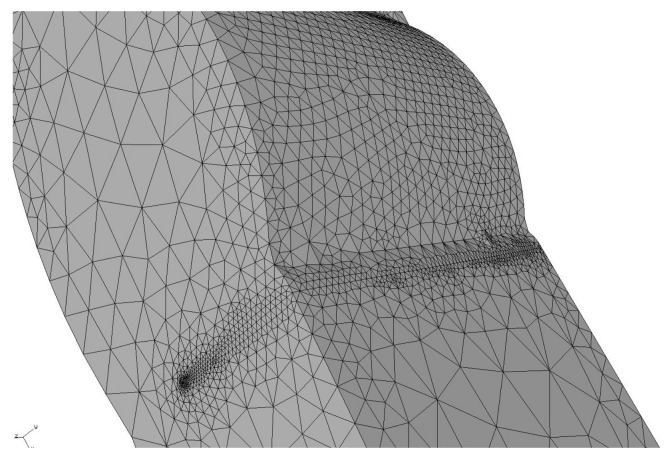
Appearance of the crack for the model with 3 mm of weld reinforcement.

Figure 11 shows the crack’s view when it reaches its critical size for the model, along a 3 mm of weld over thickness.

**Figure 12:**
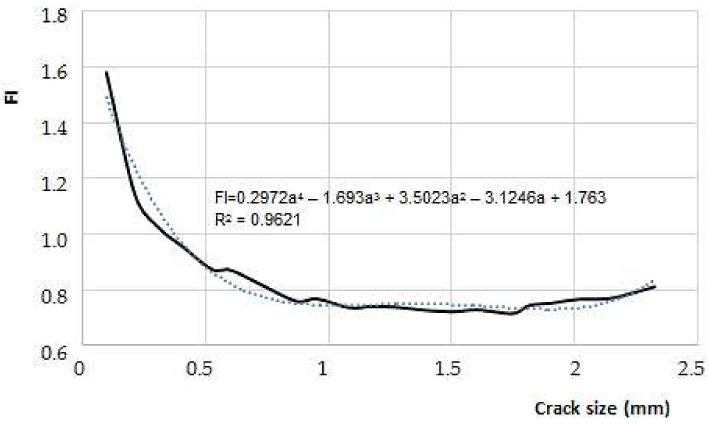
Behavior of F_I_ as a function of the crack depth (for an over thickness of 1 mm).

**Figure 13:**
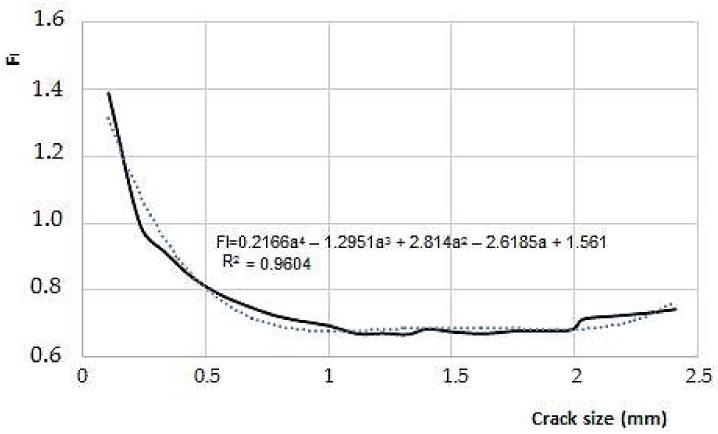
Behavior of F_I_ as a function of the crack depth (for an over thickness of 3 mm).

Figure 12 and Figure 13 show dimensionless stress factors values reached, as a function of crack size for the two models used. 

On the other hand, behavior of K_I_ indicates there is a stabilization in its length along the crack; as it grows a little, the formation of two peaks for K_I_ begins. It is located next to the ends of the crack. However, whenever there was a change on the propagation surface, as in the case with the weld bead on a thickness of 3 mm, the magnitude of the stress intensity factor at the crack tip grows in comparison to the test piece of 1mm, whose crack spread on the surface only.

**Figure 14:**
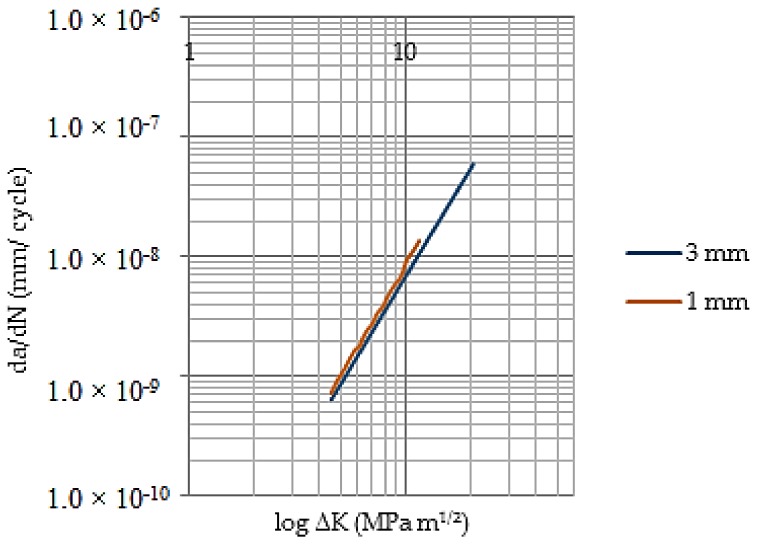
Propagation crack rate versus variation range for SIF.

Using Paris’s model, it was possible to state the stable growth rate for the two models. Figure 14 shows the behavior of the crack spread rate depending on the variation range of the stress intensity factor for both models. There have been used typical values of a ferritic–steel for C = 6.89 × 10^−12^ and *n* = 3, when the da/dN are expressed in m/cycle and ΔK in MPa m^1/2^.

#### 3.2.3. Experimental Results for Parameter on Paris’ Expression

**Figure 15:**
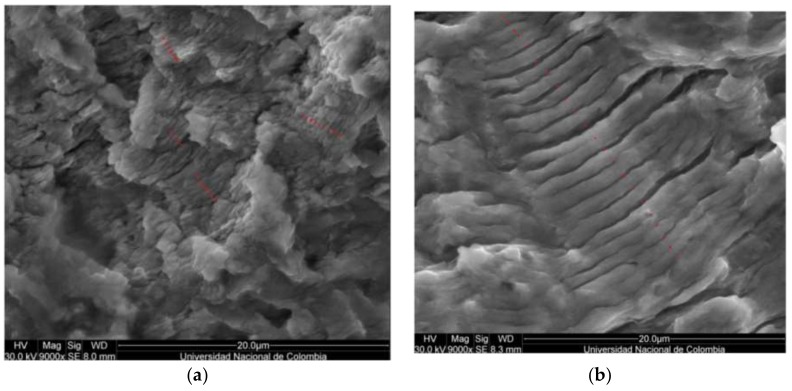
Striation counting close to the crack origin and close to the end of stable crack propagation for specimen 3F-H. (**a**) Position 0–0.001 m, 3.48 × 10^−7^ m/cycle (9000×); (**b**) Position 0.004–0.0054 m, 1.17 × 10^−6^ m/cycle (9000×).

When crack propagation rates are measured at beginning of stable zone and close to its end then the parameters of the expression of Paris can be obtained. The crack propagation rates for both positions are found using SEM by means of separation measurement between striations [16]. In Figure 15a,b, the striations counting performed on sample 3F-H is shown. In addition, the SIF range is calculated using the adjusted finite element model. Then, C and m are obtained by:

(3) $$C=\frac{{\left(\frac{\mathrm{da}}{\mathrm{dN}}\right)}_{1}}{{\left(\Delta K_{I,1}\right)}^{m}}$$

(4) $$m=\frac{\log{\left(\frac{\mathrm{da}}{\mathrm{dN}}\right)}_{1}-\log{\left(\frac{\mathrm{da}}{\mathrm{dN}}\right)}_{2}}{\log\Delta K_{I,1}-\log\Delta K_{I,2}}.$$

In Table 8, the most representative results for C and m are shown. In some of the fatigue surfaces it is not possible to establish the distance among striations, because these are not visible or are confusing among other superficial details. It is observed that maximum value for the propagation rate of the cracks is for sample 1S-G with 1.340 × 10^−6^ m/cycle, while the sample 1F-H presents a maximum value of 7.970 × 10^−7^ m/cycle, much lower than the previous one. The latter is consistent with experimental results where fatigue life for the sample 1S-G and 1F-H are 898,000 cycles and 245,000 cycles, respectively.

### 3.3. Other Mechanical Property Results

The average results of tension test for each treatment are shown in Table 9. The differences among treatments are not rather significant in means of tension strength and elastic limit. It is important to note the area under the curve for treatment 1F in contrast with treatment 3S present a well-defined difference. This indication allows one to state that, regardless of the post welding cooling medium, specimens with 1 mm of welding reinforcement will have a higher resistance to crack propagation than specimens with 3 mm of welding reinforcement, as it is observed in results from S-N curve and the surviving probability from Figure 6 and Figure 7, respectively.

**Figure 16:**
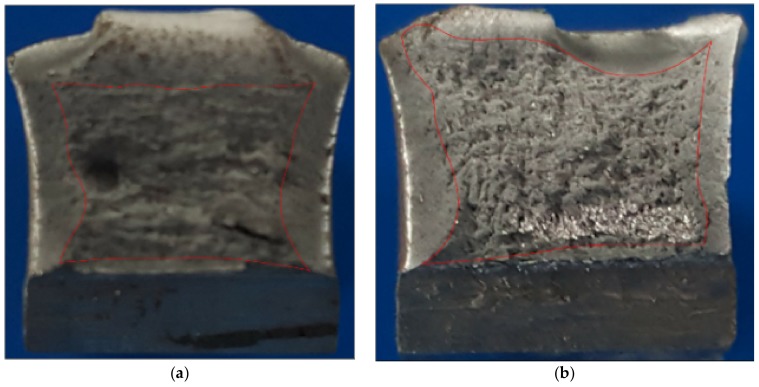
Comparison of shear fracture appearance between two specimens, (**a**) 1S-CI-HAZ (56%) and (**b**) 3F-CI-FZ (21%).

The mean results for the Charpy impact energy test is shown in Table 10. The fracture toughness of the material was calculated indirectly with the Rolfe-Barsom correlation [32]. In Figure 16, it is presented a comparison between the percent shear fracture for the highest and lowest percentages obtained by following the standard ASTM A370-14 [33], which suggests that percent shear is proportional to material ductility.

**Figure 17:**
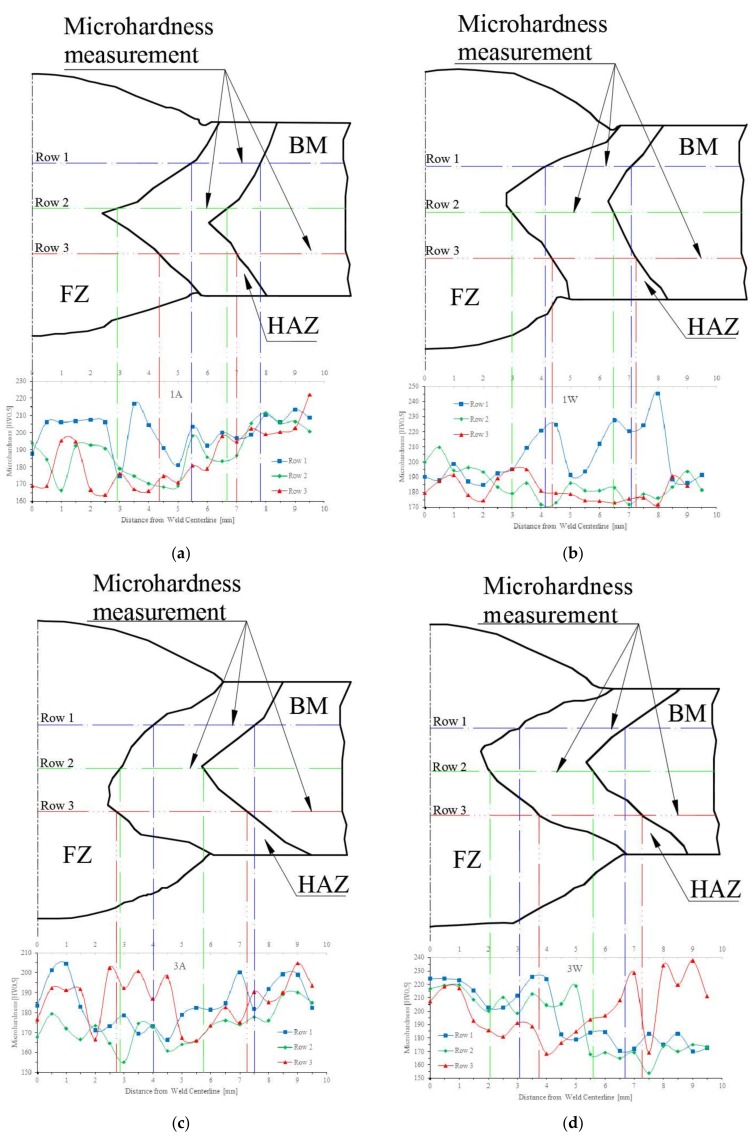
Microindentation hardness profiles for specimens of each treatment: (**a**) specimen 1S; (**b**) specimen 1F; (**c**) specimen 3S; and (**d**) specimen 3F.

The results obtained from microindentation hardness testing are shown in Figure 17, for the four studied treatments. The presented profiles are the real ones. These were obtained by means of digitalization of macro structure image and later processing on CAD software. A strong tendency to present softness for zone HAZ is presented in these results, due to the coarse grains, even though in samples 1F and 3F this behavior is present in a more noteworthy way. Although in Figure 17a–d, the hardness increase is observed on rows 1 and 3 for the samples 1F and 3F, respectively, the hardness value increases for sample 1F on the interfaces FZ-HAZ and HAZ-BM, while in sample 3F that happens on the interfaces HAZ-BM. The possible reasons for the previous behavior could be attributed to fast cooling rate and number of welding applied passes, thus generating microstructural variations in size and shape of the grains.

**Figure 18:**
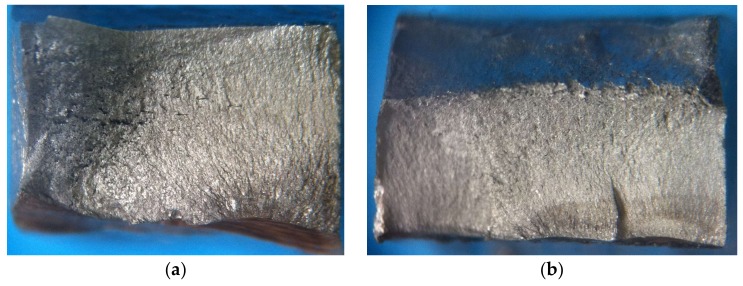
Stereo microscopy of fatigue surfaces. Sample 1S-G, with 898,000 cycles to failure (**a**) and sample 3S-E, with 75,000 cycles to failure (**b**).

In Figure 18, a comparison between samples 1S-G and 3S-E is carried out in terms of appearance of the failure zone due to fatigue. It is observed that sample 1S-G presents a single visible crack origin due to the radial marks point toward a single direction, in opposition to sample 3S-E contains three dots which indicate the crack origin of superficial semielliptical kind, based on Figure 4. In sample 3S-E (Figure 18b), it is clearly observed how the three crack fronts, as crack propagation move along, tend to convert into a single crack front, while in sample 1S-G (Figure 18a) the only crack front present propagates nearly in a concentric way around its origin. The bigger size of stable crack growth for a same load cycle means a higher tolerance to damage of the welded joint.

Finally, the test specimens dimensionally stayed with the tolerances required using abrasive waterjet (AWJ) this manufacturing process did not affect the mechanical properties on the lateral surfaces since all the specimens failed due to cracks that started from weld toe. Therefore, the effect of the machining process on experimental fatigue results is ruled out. In the research [34], as in the present research, no dimensional deviations were detected using this manufacturing procedure. 

## 4. Conclusions

According with the results experimentally obtained in this research, welded joints with a welding reinforcement of 1 mm and cooled in water reached the highest probability of survival to fatigue. While the welded joints with a welding reinforcement of 3 mm and cooled in calm air, reached the worst probability of survival to fatigue. In general, fatigue strength in butt welded joints is strongly influenced by the welding reinforcement. In this research, 93.8% of the specimens presented a fatigue strength superior to the one of class FAT 90, while about 78.1% of the specimens reached a fatigue strength higher than the recommended one by class FAT 100.

The use of the finite element method, along with the fracture mechanics, allowed one to have an adequate simulation of the stable propagation of cracks. The integration of the results obtained for the variation range for stress intensity factor and the separation between striations, obtained through SEM observation, allowed the estimation of the parameters C and m from Paris’ expression. For some measurements, C values and m atypical were obtained. The latter could be due to the high gradient change for the local mechanical properties next to the welding bead and the inherent distance difficulties of the measuring method between striations. However, the numerical propagation rates in the order of those found through experimental measurements were obtained.

The crack front on the failure surface presented an approximated semielliptical shape. This allows one to establish the finite element modelling for butt welded joints to establish correlations between experimental results and theoretical calculations. On the other hand, the importance of performing additional studies where the crack nucleation stage is included is highlighted. Where a fatigue model for this first stage should cover the influence of the microstructural phases and the grains shape and size. Moreover, further improvement of this assessment is expected by implementing other specific models for predicting changes in the microstructure and mechanical properties during the thermal cycle of the welding process.

In consistency with welding and post-welding processes applied to butt welded joints, it was observed that the post welding cooling cycle and the quantity of weld passes influence the material ductility and, thus the fracture toughness of the material, as well. In general, higher post-welding cooling speed caused significant lower measured values for the Charpy impact energy. Lower values of the Charpy impact energy are reported for the FZ and the HAZ compared to the base metal. On the other hand, no significant differences were found between experimental treatments for the mechanical properties obtained by the tension tests.

## Figures and Tables

**Table 1:** Coding used for the uniaxial fatigue test specimens.

Code	Experimental Factors		Number of Replicas
	Welding Reinforcement (mm)	Post-Welding Cooling Medium	
1S-(replica)	1	Quiet air	8
1F-(replica)	1	Water	8
3S-(replica)	3	Quiet air	8
3F-(replica)	3	Water	8

**Table 2:** Coding used for the Charpy impact energy test specimens.

Code	Experimental Factors			Number of Replicas
	Welding Reinforcement (mm)	Post-Welding Cooling Media	Impact Zone Located at	
1S-FZ-(replica)	1	Quiet air	Fusion zone	3
1S-HAZ-(replica)	1	Quiet air	Heat affected zone	3
1F-FZ-(replica)	1	Water	Fusion zone	3
1F-HAZ-(replica)	1	Water	Heat affected zone	3
3S-FZ-(replica)	3	Quiet air	Fusion zone	3
3S-HAZ-(replica)	3	Quiet air	Heat affected zone	3
3F-FZ-(replica)	3	Water	Fusion zone	3
3F-HAZ-(replica)	3	Water	Heat affected zone	3
BM	-	-	Base metal	3

**Table 3:** Coding used for the uniaxial tension test specimens.

Code	Experimental Factors		Number of Replicas
	Welding Reinforcement (mm)	Post-Welding Cooling Medium	
1ST-(replica)	1	Quiet air	3
1FT-(replica)	1	Water	3
3ST-(replica)	3	Quiet air	3
3FT-(replica)	3	Water	3

**Table 4:** Mechanical properties for ASTM A36 steel.

Yield Stress (MPa)	Ultimate Tensile Strength (MPa)	Elongation (%)
262	434	38

**Table 5:** Chemical composition for ASTM A36 steel, (wt. %).

Chemical Composition	Weight Percentage (wt. %)
C	0.202
Mn	0.532
Si	0.030
P	0.028
S	0.011

**Table 6:** Crack sizes of semi elliptical section using each iteration.

Semi-Major Axis (c) (mm)	Semi-Minor Axis (a) (mm)
0.15	0.06
0.23	0.09
0.30	0.12
0.45	0.18
1.25	0.50
2.50	1.00
5.00	2.00
7.50	3.00

**Table 7:** Relative error and computational cost on determining K_I_.

Models	Nodes	Elements	Time CPU (s)	K_I_ $\left(MPa\sqrt{m}\right)$	% Error
Anderson’s analytical models	-	-	-	4.41	-
Numerical model 1	68 426	41 758	45	4.34	1.62
Numerical model 2	63 124	38 294	35	4.09	7.04
Numerical model 3	49 892	30 260	25	4.13	6.40

**Table 8:** Summary of the parameters calculation of Paris expression for several samples.

Specimen	a_1_(m)	a_2_(m)	${\left(\frac{da}{dN}\right)}_{1}$ (m/cycle)	${\left(\frac{da}{dN}\right)}_{2}$ (m/cycle)	∆K_I,1_ ($MPa\;\sqrt{m}$)	∆K_I,2_ ($MPa\;\sqrt{m}$)	C	m
1S-G	9.00 × 10^−4^	2.45 × 10^−3^	6.280 × 10^−7^	1.341 × 10^−6^	10.2	16.9	6.14 × 10^−8^	1.51
1F-H	2.40 × 10^−3^	5.29 × 10^−3^	2.341 × 10^−7^	7.974 × 10^−7^	16.7	24.8	1.40 × 10^−8^	3.10
3S-D	1.09 × 10^−3^	5.41 × 10^−3^	5.411 × 10^−7^	1.280 × 10^−6^	11.2	25.1	4.81 × 10^−8^	1.08
3F-H	6.50 × 10^−4^	4.42 × 10^−3^	3.482 × 10^−7^	1.141 × 10^−6^	8.7	22.7	4.01 × 10^−8^	1.24

**Table 9:** Results for the mechanical properties obtained by uniaxial tensile tests (average values).

Specimen	Ultimate Tensile Strength (MPa)	Yield Stress 0.20% (MPa)	Percent Reduction of Area (%)	Elongation (%)
1S	462	332	31.4	19.4
1F	461	332	32.1	23.3
3S	455	322	32.1	18.3
3F	465	322	28.9	19.6

**Table 10:** Results of Charpy impact energy tests (average values).

Specimen	CVN (J)	K_Ic_ ($MPa\;\sqrt{m}$)	Shear Fracture (%)	Specimen	CVN (J)	K_Ic_ ($MPa\;\sqrt{m}$)	Shear Fracture (%)
1S-FZ	100.29	163.3	33	1S-HAZ	157.04	157.4	56
1F-FZ	81.17	146.2	33	1F-HAZ	138.67	147.8	40
3S-FZ	75.97	141.2	21	3S-HAZ	125.98	140.7	45
3F-FZ	66.91	131.9	21	3F-HAZ	187.01	172.0	31
BM	204.63	180.0	27	-	-	-	-
